# A thymus-specific noncoding RNA, Thy-ncR1, is a cytoplasmic riboregulator of MFAP4 mRNA in immature T-cell lines

**DOI:** 10.1186/1471-2199-11-99

**Published:** 2010-12-16

**Authors:** Kazuma Aoki, Akira Harashima, Miho Sano, Takahide Yokoi, Shuji Nakamura, Masayoshi Kibata, Tetsuro Hirose

**Affiliations:** 1 Functional RNomics Team, Biomedicinal Information Research Center, National Institute of Advanced Industrial Science and Technology (AIST) 2-4-7 Aomi, Koutou, Tokyo 135-0064, Japan; 2 Japan Biological Informatics Consortium (JBIC), 2-4-7 Aomi, Koutou, Tokyo 135-0064, Japan; 3 Hayashibara Biochemical Labs, Inc., 675-1 Fujisaki, Okayama 702-8006, Japan; 4 Hitachi Software Engineering, Ltd., 1-1-43 Suehirocho, Tsurumi, Yokohama 230-0045, Japan

## Abstract

**Background:**

Postgenomic transcriptome analyses have identified large numbers of noncoding (nc)RNAs in mammalian cells. However, the biological function of long ncRNAs in mammalian cells remains largely unknown. Our recent expression profiling of selected human long ncRNAs revealed that a majority were expressed in an organ-specific manner, suggesting their function was linked to specific physiological phenomena in each organ. We investigated the characteristics and function of ncRNAs that were specifically expressed in the thymus, the site of T-cell selection and maturation.

**Results:**

Expression profiling of 10 thymus-specific ncRNAs in 17 T-cell leukemia cell lines derived from various stages of T-cell maturation revealed that HIT14168 ncRNA, named Thy-ncR1, was specifically expressed in cell lines derived from stage III immature T cells in which the neighbouring CD1 gene cluster is also specifically activated. The Thy-ncR1 precursor exhibited complex alternative splicing patterns and differential usage of the 5' terminus leading to the production of an estimated 24 isoforms, which were predominantly located in the cytoplasm. Selective RNAi knockdown of each Thy-ncR1 isoform demonstrated that microfibril-associated glycoprotein 4 (MFAP4) mRNA was negatively regulated by two major Thy-ncR1 isoforms. Intriguingly, the MFAP4 mRNA level was controlled by a hUPF1-dependent mRNA degradation pathway in the cytoplasm distinct from nonsense-mediated decay.

**Conclusions:**

This study identified Thy-ncR1 ncRNA to be specifically expressed in stage III immature T cells in which the neighbouring CD1 gene cluster was activated. Complex alternative splicing produces multiple Thy-ncR1 isoforms. Two major Thy-ncR1 isoforms are cytoplasmic riboregulators that suppress the expression of MFAP4 mRNA, which is degraded by an uncharacterized hUPF1-dependent pathway.

## Background

Recent postgenomic transcriptome analyses, including cDNA sequencing and tiling array analyses, have revealed that large numbers of transcripts unlikely to encode polypeptides are produced from regions covering a large fraction of the human and mouse genomes [1-6]. A recent report from the ENCODE project [4] estimated that 93% of the human genome is transcribed into RNA, whereas only 2% of the human genome codes for protein [7]. Therefore, most of the RNAs transcribed from the human genome must be non-protein coding transcripts, commonly called noncoding RNAs (ncRNAs).

The limited number of long ncRNAs that have been characterized to date exhibit diverse functions, as well as cell type-specific expression and localization to subcellular compartments. Further determination of the functions of long ncRNAs will expand our understanding of various fundamental biological processes. Many recent efforts in transcript mapping and expression profiling have provided a rough overview of putative ncRNA functions. A major subset of ncRNAs associates with specific chromosomal loci, where they may play a role in altering chromosomal structure and regulating gene expression. The Xist, Air, and Kcnq1ot1 ncRNAs are involved in genomic imprinting accompanied by structural changes to the chromosome [8-10]. HOTAIR has been shown to recruit the PRC2 histone modification complex, regulate specific HOX gene loci on different chromosomes [11], and reprogram chromosomal structure in a manner that leads to cancer metastasis [12]. Furthermore, the PRC2 complex was found to associate with hundreds of large intergenic non-coding RNAs (lincRNAs) that were identified by a genome-wide search based on the histone H3K36 code [13]. These data indicate a general long ncRNA function in epigenetic control of gene expression. Additionally, recent reports have indicated that a subset of ncRNAs play essential roles in intracellular substructure organization. The MENε/β ncRNAs are essential for nuclear paraspeckle formation through their interaction with specific RNA-binding proteins [14-16].

In contrast to their nuclear functions, little is known about the cytoplasmic functions of long ncRNAs in mammalian cells. The NRON ncRNA was reported to associate with importin β in the cytoplasm and may regulate the nuclear transport of the NFAT transcription factor upon calcium signaling [17]. The UHG and gas5 ncRNAs, which produce small nucleolar RNAs (snoRNAs) from their introns, are transported into the cytoplasm where they are rapidly degraded by nonsense-mediated decay (NMD) [18-20]. Therefore, the spliced exons of UHG and gas5 may be nonfunctional transcripts. However, the gas5 ncRNA is involved in the proliferation of a leukemia cell line, and a fragment of the gas5 ncRNA acts as a riboregulator of a nuclear receptor [21,22]. In plants, the ncRNA IPS1 acts as a molecular decoy for a specific miRNA and attenuates the effect of that miRNA on its target mRNA [23].

Cytoplasmic mRNA levels are controlled via various mRNA degradation pathways that recognize the structural features of mRNAs targeted for degradation. Aberrant mRNAs with a premature termination codon (PTC) are specifically recognized by RNA surveillance machinery and committed to the NMD pathway [19]. The NMD pathway also degrades UHG and gas5 ncRNAs transported into the cytoplasm [18]. In mammalian cells, hUPF1 triggers NMD of PTC-containing mRNAs or ncRNAs and interacts with hUPF2, which associates with the exon junction complex (EJC) on spliced mature mRNAs [19]. Staufen1, which binds to a specific stem-loop structure in the 3'UTR, also recruits hUPF1, and the interaction elicits the degradation of the target mRNAs [24]. hUPF1 was recently shown to interact with RISC and be involved in the destabilization of miRNA-targeted mRNAs [25].

Here, we identified long ncRNAs that are expressed specifically in the thymus. The thymus is an organ located in the upper anterior portion of the chest and is the site of T-lymphocyte maturation. Therefore, thymus-specific ncRNAs are intriguing candidates for factors involved in the regulation of thymus-specific physiological events such as T-lymphocyte maturation. One thymus-specific ncRNA, Thy-ncR1, was expressed only in a few T-cell lines, all of which originated from immature stage III T cells. Thy-ncR1 exhibits complex alternative splicing to produce several isoforms. We further identified a putative target mRNA that is controlled by Thy-ncR1 via a novel hUPF1-dependent RNA degradation pathway.

## Results

### The thymus-specific ncRNA, Thy-ncR1, is expressed in a subset of leukemia T-cell lines

To identify long ncRNAs that are involved in thymus-specific physiological events, we examined a microarray dataset obtained using our original microarray to monitor the expression of human non-protein coding transcripts selected from the H-Invitational database http://www.h-invitational.jp/hinv/ahg-db/index.jsp[26]. ncRNA-like transcripts that were predominantly expressed in the thymus were selected based on a simple comparison of the microarray signals from human thymus, brain, liver, and testis.

**Figure 1:**
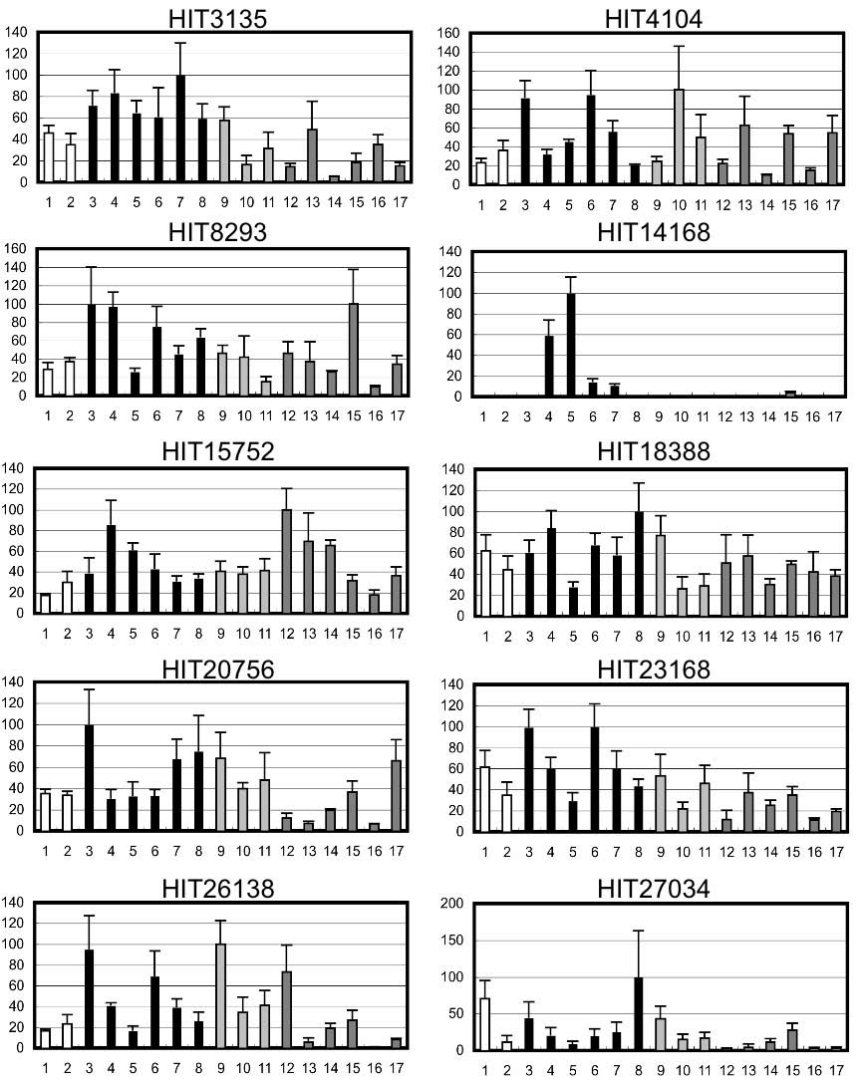
**Expression profiles of thymus-specific ncRNAs in T-cell leukemia cell lines**. Quantitation of each ncRNA by qRT-PCR is shown in the graph. The expression level of the cell line showing the highest expression was defined as 100%. Bars represent the means ± SD of three measurements. The following T-cell leukemia cell lines were used: 1, RPMI-8402; 2, H-SB2; 3, CCRF-CEM; 4, DND41; 5, HPB-ALL; 6, Jurkat; 7, MOLT-3; 8, TALL-1; 9, MOLT-13; 10, MOLT-16; 11, PEER; 12, HUT-78; 13, MOTN-1; 14, MT-1; 15, SKW-3; 16, HUT-102; and 17, MT-2. The cell lines are categorized into four types according to EGIL criteria (7). Type-T-II includes cell lines #1 and 2 (open graph bars); type-T-III includes lines #3 to #8 (black graph bars); type-T-IV includes lines #9 to #11 (light gray graph bars); and mature T includes lines #12 to #17 (dark gray graph bars).

The thymus-specificity of each transcript was validated by quantitative reverse transcription polymerase chain reaction (qRT-PCR) using total RNA samples prepared from 11 human organs, including the 4 listed above (Additional file 1 Fig. S1). We selected 10 thymus-specific ncRNAs that included three ncRNAs identified in our primary screening of tissue-specific ncRNAs [27]. For further characterization of the expression pattern of each ncRNA during T-cell maturation in the thymus, 17 T-cell leukemia cell lines were examined. These leukemia cell lines were originally derived from T cells at various stages of maturation, and each cell line therefore partially reflects the features of native T-cell maturation. The cell lines were used for expression profiling of the 10 thymus-specific ncRNAs, and qRT-PCR analyses revealed that the majority of the thymus-specific ncRNAs were expressed in all 17 T-cell leukemia cell lines (Figure 1). However, the expression of HIT14168 was restricted to the T-cell lines DND41, HPB-ALL, Jurkat, and MOLT-3, which are all derived from immature stage III T cells [28,29] (Figure 1). This specific expression pattern suggests that the HIT14168 ncRNA functions in some physiological process specific to immature stage III T cells. We further characterized this transcript, which we have named thymus-specific ncRNA-1 (Thy-ncR1).

### The Thy-ncR1 gene is coordinately expressed with the CD1 gene cluster in a cell lineage-specific manner

The Thy-ncR1 gene is located at 1q23.1 on human chromosome 1. This locus overlaps with a number of olfactory receptor (OR) genes. Indeed, an OR10R2 gene and a related OR pseudogene are encoded in the antisense strand of the Thy-ncR1 gene. Since OR gene expression is normally restricted to the olfactory bulb, the thymus-specific Thy-ncR1 is unlikely to control OR genes. However, the possibility that this subset of OR genes is expressed during T-cell maturation and that Thy-ncR1 acts as a regulatory antisense RNA could not be excluded. Therefore, the possible regulatory role of Thy-ncR1 in OR10R2 gene expression was first examined in the thymus and in the Jurkat T-cell leukemia cell line. An RNase A/T1 protection assay using an RNA probe covering the OR10R2 gene in exon 2 of Thy-ncR1 revealed that OR10R2 gene expression was undetectable both in thymus tissue and Jurkat cells, whereas Thy-ncR1 was highly expressed (Additional file 1, Fig. S2A and S2B). This result strongly suggests that Thy-ncR1 has a T-cell-specific function(s) independent of OR gene expression.

**Figure 2:**
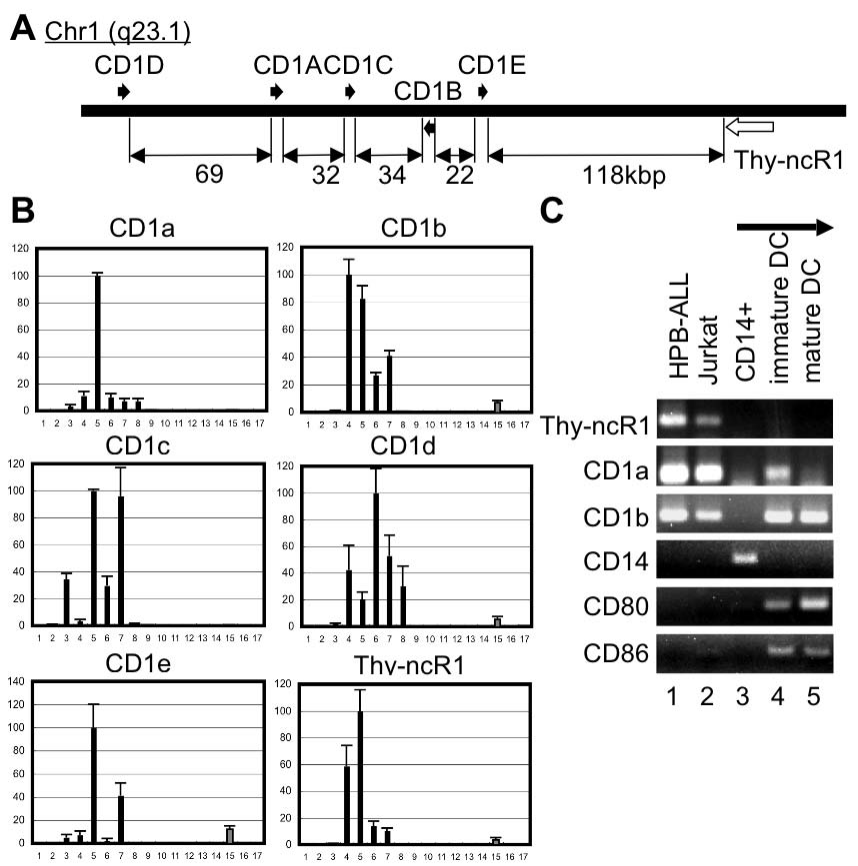
**Coordinated expression of Thy-ncR1 and CD1 gene cluster**. A. Genomic location of Thy-ncR1 on chromosome 1 (q23.1). The CD1 gene cluster is adjacent to Thy-ncR1. The distance between each gene is shown below the diagram in kbp. B. The expression of the CD1 gene family is highly correlated to Thy-ncR1 expression. Quantitation of each gene transcript by qRT-PCR is shown in the graph. The highest expression level among the 17 cell lines was defined as 100% for each transcript. Bars represent means ± SD for three independent experiments. C. Thy-ncR1 expression is cell-lineage specific. RT-PCR to detect Thy-ncR1, the CD1a and 1b gene transcripts, and the DC differentiation markers CD14, CD80, and CD86.

The T-cell-related CD1 gene cluster is located at an adjacent chromosomal locus, 118 kb from the Thy-ncR1 gene (Figure 2A). CD1 is a classical cell surface marker antigen, which displays lipid antigens on the surface of dendritic cells; however, its exact function in T cells remains elusive [30]. Expression profiling of CD1 gene family members (CD1a-1e) in the 17 T-cell leukemia cell lines revealed that their expression correlated with that of Thy-ncR1 (Figure 2B). Expression of CD1a, 1b, 1c, and 1e, which are class I CD1 proteins and more proximal to Thy-ncR1, almost completely overlapped with Thy-ncR1 expression with respect to both the cell lines in which CD1a, 1b, 1c, and 1e were expressed and their relative expression levels in those cell lines (Figure 2B). CD1 d, the only class II CD1 protein in the cluster, was expressed with a similar pattern but more variably than the class 1 CD1 genes (Figure 2B).

In rodents, class I CD1 genes have been lost from the genome, resulting in only the class II CD1 d gene remaining at the syntenic locus [31] (additional file 1, Fig. S3). The search for a putative Thy-ncR1 ortholog near the CD1 d gene and at other loci in the mouse genome by sequence comparison was unsuccessful, suggesting that Thy-ncR1 was lost from the mouse genome with the class I CD1 genes (Additional file 1, Fig. S3). Thus, Thy-ncR1 may play a role in cooperation with class I CD1 proteins in immature T cells or, alternatively, control class I CD1 gene expression in humans.

CD1 genes are expressed in dendritic cells (DC) as well as in T cells. Therefore, the coordinated expression of Thy-ncR1 and CD1 genes was then investigated in the DC lineage. The expression of Thy-ncR1 as well as CD1a and CD1b was monitored during differentiation of DCs from CD14^+ ^monocytic cells [32]. In contrast to T cells, Thy-ncR1 was not expressed during DC differentiation, although the CD1 genes were expressed (Figure 2C). This result clearly demonstrated that the coordinated expression of Thy-ncR1 with the class 1 CD1 genes is T-cell lineage-specific.

### Complex alternative splicing produces multiple Thy-ncR1 isoforms

**Figure 3:**
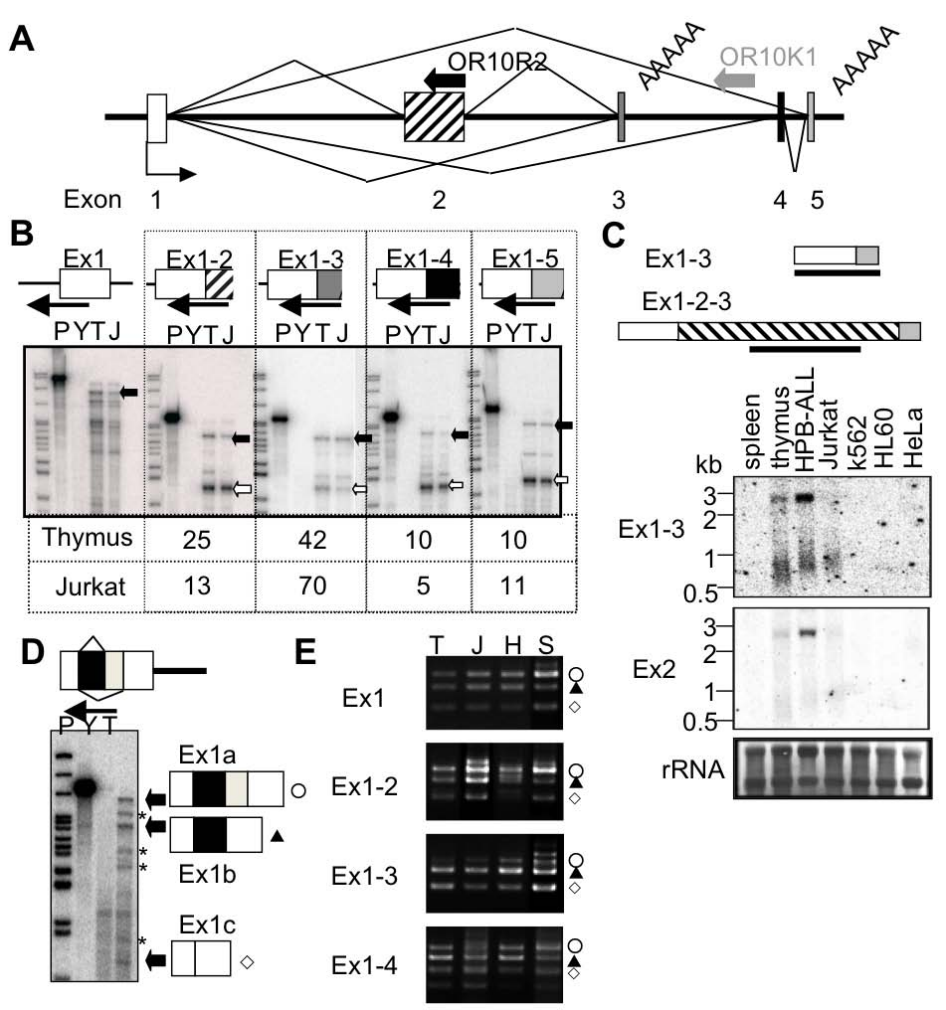
**Complex alternative splicing to produce multiple Thy-ncR1 isoforms**. A. Schematics of Thy-ncR1 isoform formation by alternative splicing. The polyadenylation sites are shown. The olfactory receptor gene OR10R2 and a pseudogene (OR10K1) encoded in the antisense strand are shown by the bold arrows. The exon numbers are shown below the diagram. B. The relative amounts of Thy-ncR1 isoforms. An RNase protection assay using riboprobes spanning the exon-exon junction of each isoform was conducted using total RNA from thymus (T) and Jurkat (J) cells. The negative control was yeast RNA (Y). The bands corresponding to each spliced isoform and the other isoforms in terms of each riboprobe are shown by closed and open arrows, respectively. The ratio of each isoform in thymus and Jurkat cells, which was calculated by dividing the band intensity of each isoform (closed arrow) by the sum of the band intensities of all isoforms (closed arrow + opened arrow), is shown below. C. Northern hybridization analyses to detect Thy-ncR1 isoforms. The DNA probes used are shown above. D. Additional splicing variants within exon 1. A schematic diagram of alternative splicing is shown above. RNase protection assay probe I derived from exon 1 is indicated by an arrow. The patterns obtained by RNase protection assays are shown in the lower panel with a diagram for each isoform (Ex 1a, 1b, and 1c). E. The combination formed by each exon 1 isoform spliced to other exons. RT-PCR using the exon 1 primer that detects all exon 1 isoforms, and an exon 2, 3, or 4 primer was carried out with RNA prepared from thymus (T), HPB-ALL (H), Jurkat (J), and SKW3 (S) cells. Three symbols (circle, triangle, and square) correspond to the exon 1 isoforms shown in D.

According to the H-Invitational database, the Thy-ncR1 transcript (HIT14168) is estimated to be 2.6 kb in size and composed of three exons. In our previous study, northern hybridization to detect HIT14168 detected the accumulation of a ~0.7-kb transcript in the thymus [27]. These data indicate that the Thy-ncR1 gene produces several ncRNA isoforms, presumably by alternative splicing. cDNA cloning based on EST data revealed the existence of at least 4 different splicing isoforms, Ex1-2-3 (= HIT14168), Ex1-3, Ex1-4-5, and Ex1-5, in the thymus (Figure 3A). RNase protection assays using probes covering the exon-exon junction of each splicing isoform revealed that Ex1-3 was the most abundant isoform in both thymus tissue (42%) and T-cell leukemia Jurkat cells (70%). Ex1-3 is estimated to be 0.7 kb, corresponding to the transcript previously detected by northern hybridization analysis [27]. Northern hybridization analysis with a probe corresponding to Ex1-3 detected a ~0.7-kb transcript in the thymus and in the HPB-ALL and Jurkat T-cell leukemia cell lines, whereas the Ex2 probe detected a 2.6-kb transcript that corresponds to Ex1-2-3 with a similar expression specificity (Figure 3C). The broad band corresponding to Ex1-3 (~0.7 kb) could be sharpened by RNase H treatment in the presence of oligo dT, indicating that a heterogeneous population of the Ex1-3 transcript possessing poly(A) tails exists (data not shown). Two Thy-ncR1 3' termini were identified by 3' RACE downstream of the canonical poly (A) signals mapped at the ends of exon 3 and exon 5 (Figure 3A).

An RNase protection assay using the Ex1 probe (probe I) detected several bands in addition to one corresponding to the full-length exon 1 (Figure 3D and additional file 1, Fig. S2C), suggesting that additional alternative splicing sites are present within exon 1. Further cDNA cloning and RNase protection assay of the exon 1 region revealed that two additional splice sites existed in exon 1, which resulted in the production of three exon 1 isoforms: exon 1b, exon 1c, and the unspliced exon (exon 1a) (Figure 3D). The additional exon 1-derived forms are shown in Figure 3D (asterisks). Another RNase protection assay probe lacking 135 nt of the exon 1 5' terminus did not detect these bands (probe II in Additional file 1, Fig. S2), suggesting that these transcripts correspond to isoforms whose 5' end is shifted downstream. Taken together, the total number of Thy-ncR1 splicing isoforms is estimated to be at least 24 (12 produced by variations in exon combinations and an additional 12 produced by the 5' end heterogeneity).

Next, we investigated the levels of the three exon 1 isoforms linked to downstream exon 2, 3, or 4 by RT-PCR using primer pairs for the exon 1 common primer and various exon 2-4 specific primers (Figure 3E). Exon 1a was most frequently combined with exon 2 (Figure 3E, open circles), whereas exons 3 and 4 were more frequently combined with exon 1b (Figure 3E, closed triangles). The level of the exon 1c-3 isoform was remarkably increased in SKW3 cells compared to the other three T-cell lines (Figure 3E, open squares). In contrast, exon 1c was rarely combined with exon 2 and exon 4 in HPB-ALL cells (Figure 3E, bottom panel). These results indicate that the exon combinations are cell-type specific.

### Thy-ncR1 transcripts are cytoplasmic but not susceptible to NMD

**Figure 4:**
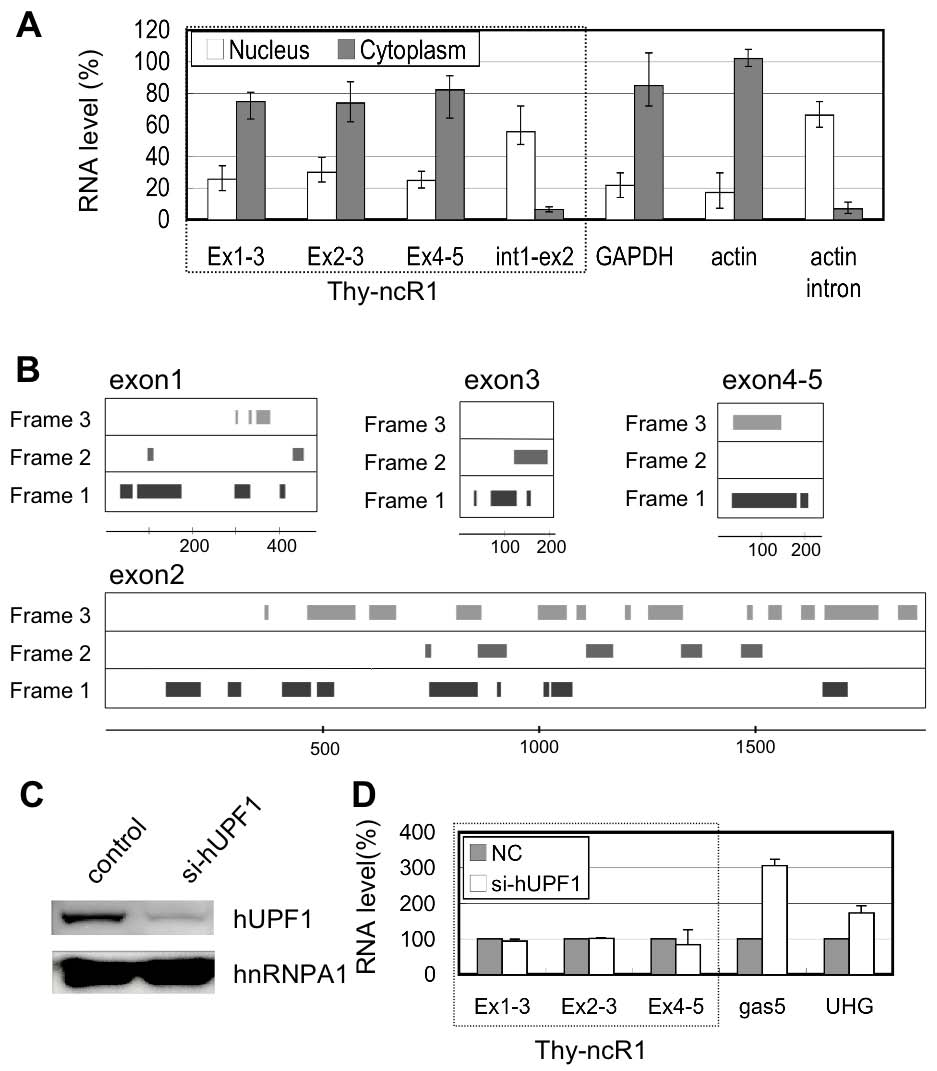
**Thy-ncR1 transcripts are cytoplasmic but are not susceptible to NMD**. A. Subcellular distribution of Thy-ncR1 isoforms. HPB-ALL cells were fractionated into nuclear and cytoplasmic fractions. RNA prepared from each fraction was used for qRT-PCR analyses. The levels of Thy-ncR1 isoforms (exon 1-3, 2-3, and 4-5) and precursor retaining intron 1 (int1-ex2) were quantified in the nuclear and cytoplasmic fractions. GAPDH and β-actin mRNA are the controls for cytoplasmic RNA, and β-actin pre-mRNA (actin intron) is the control for nuclear RNA. Thy-ncR1 is not susceptible to NMD. B. Potential ORFs (closed boxes) in Thy-ncR1 exons. The scales (nt) are shown below the diagram. C. Depletion of hUPF1 from Jurkat cells treated with siRNA (si-hUPF1) was confirmed by immunoblot analysis with anti-hUPF1 antibody. D. qRT-PCR analyses of Thy-ncR1 isoforms and known NMD targets (gas5 and UHG) in control (NC, adjusted to 100%) and hUPF1-depleted cells (si-hUPF1) as shown in B. Bars represent means ± SD for three independent experiments.

The intracellular localization of each Thy-ncR1 isoform was examined in HPB-ALL cells. The cells were homogenized and separated into nuclear and cytoplasmic fractions, followed by quantitation of Thy-ncR1 levels in each fraction by qRT-PCR. As shown in Figure 4A, the major splicing isoforms (Ex1-3, Ex1-2-3 and Ex1-4-5) were localized to the cytoplasm, similar to typical mRNAs (Figure 4A, GAPDH and β-actin). In contrast, a large fraction of the precursor Thy-ncR1, which retains intron 1 (Figure 4A, Int1-Ex2), was localized to the nucleus, similar to β-actin precursor mRNA (Figure 4A, actin intron). These results confirmed that the HPB-ALL cells were successfully fractionated and that the mature Thy-ncR1 transcript was localized to the cytoplasm. The same localization analysis was conducted in Jurkat cells, and the nucleo-cytoplasmic distribution of Thy-ncR1 isoforms was the same as that observed in HPB-ALL cells (data not shown).

The data indicating that Thy-ncR1 is cytoplasmic raised the possibility that Thy-ncR1 encodes a functional short polypeptide. Sucrose density gradient analysis revealed that Thy-ncR1 isoforms were commonly enriched in polysome-like fractions that were sensitive to EDTA treatment (Additional file 1, Fig. S4). The Thy-ncR1 isoforms were examined for ORFs, and the conservation of each ORF was determined in four primate species where putative Thy-ncR1 counterparts are predicted. In exon 1, which is included in all the isoforms, the ORF located most proximal to the 5' terminus consists of only 9 codons, and the largest ORF consists of 34 codons (see Figure 4B and Additional file 1, Fig. S5). The sequence alignment of the first (9 codons) and the second (34 codons) ORFs between the counterparts in four primates, humans, chimpanzees, orangutans, and rhesus monkeys revealed that two of the nine amino acids encoded in the first ORF were not conserved in two primate species, and a termination codon appeared at the fifth codon of the second ORF in rhesus monkeys (Additional file 1, Fig. S5). These data strongly suggest that these ORFs are unlikely to encode any functional polypeptides.

Evidence indicating that some cytoplasmic ncRNAs are degraded via the NMD pathway [18] raises the possibility that Thy-ncR1 is an NMD target. The termination codon of the ORF in the first exon would be recognized as a premature termination codon (PTC) if translated. However, RNAi depletion of hUPF1 in Jurkat cells (Figure 4C) to arrest NMD led to no obvious stabilization of any Thy-ncR1 RNA isoforms. In contrast, the levels of gas5 and UHG, both known NMD targets, were significantly elevated upon hUPF1 depletion (Figure 4D). This result indicated that Thy-ncR1 is not susceptible to NMD.

### Thy-ncR1 is a negative riboregulator of MFAP4 gene expression

**Figure 5:**
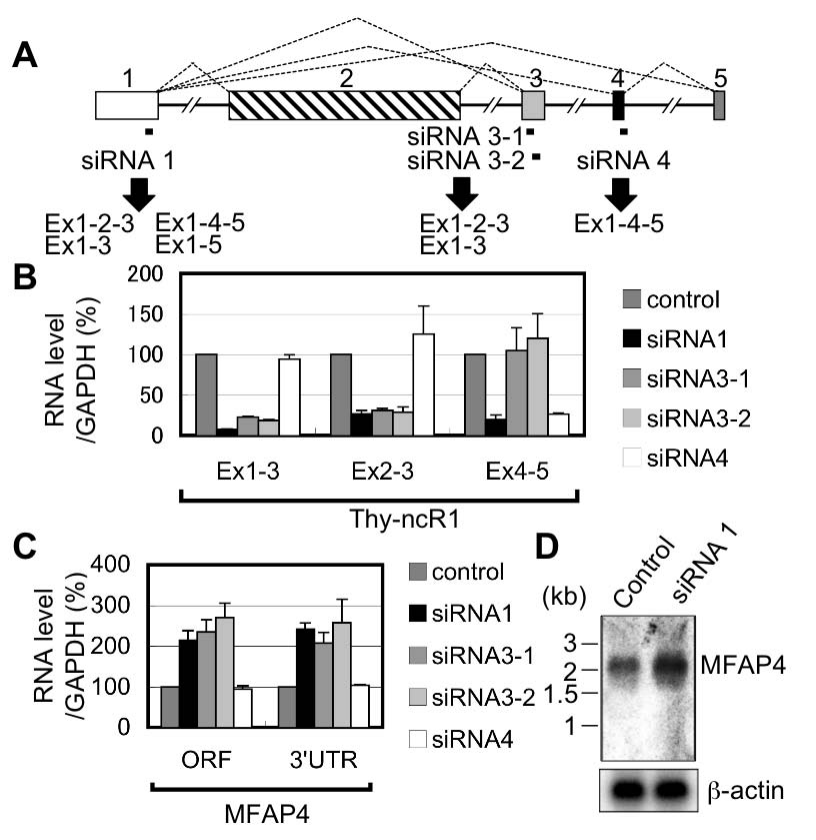
**Thy-ncR1 controls MFAP4 mRNA levels**. A. Isoform-specific knockdown of Thy-ncR1. The positions of siRNAs designed to knockdown specific Thy-ncR1 isoforms are shown. Isoforms that could be degraded by each siRNA are shown below the arrow. B. Confirmation of Thy-ncR1 knockdown. The levels of Thy-ncR1 isoforms were determined by qRT-PCR. C. Increase in MFAP4 mRNA upon Thy-ncR1 knockdown. The MFAP4 mRNA level in Thy-ncR1 knockdown cells was determined by qRT-PCR. D. RNA hybridization to detect MFAP4 mRNA in HPB-ALL cells treated with control and siRNA 1 of Thy-ncR1.

To examine the biological function of Thy-ncR1, each isoform was selectively degraded using RNAi in HPB-ALL cells. The siRNA 1, which corresponds to a sequence in exon 1, depleted all isoforms, while siRNA 3-1 and 3-2 depleted two major isoforms (Ex1-2-3 and Ex1-3), and siRNA 4 specifically depleted the Ex1-4-5 isoform (Figure 5A and 5B). Microarray analysis and subsequent qRT-PCR verification were used to identify genes whose expression was controlled by Thy-ncR1 isoforms. Microfibril-associated glycoprotein 4 (MFAP4) mRNA was elevated ~2.5-fold upon depletion of Thy-ncR1 isoforms with siRNAs 1, 3-1, and 3-2, but not siRNA 4 (Figure 5C). Northern hybridization revealed that MFAP4 mRNA accumulated as a single isoform of ~2 kb, and its level increased without any changes in mRNA length upon Thy-ncR1 knockdown (Figure 5D).

### Possible involvement of Thy-ncR1 in a novel hUPF1-dependent pathway for MFAP4 mRNA degradation

**Figure 6:**
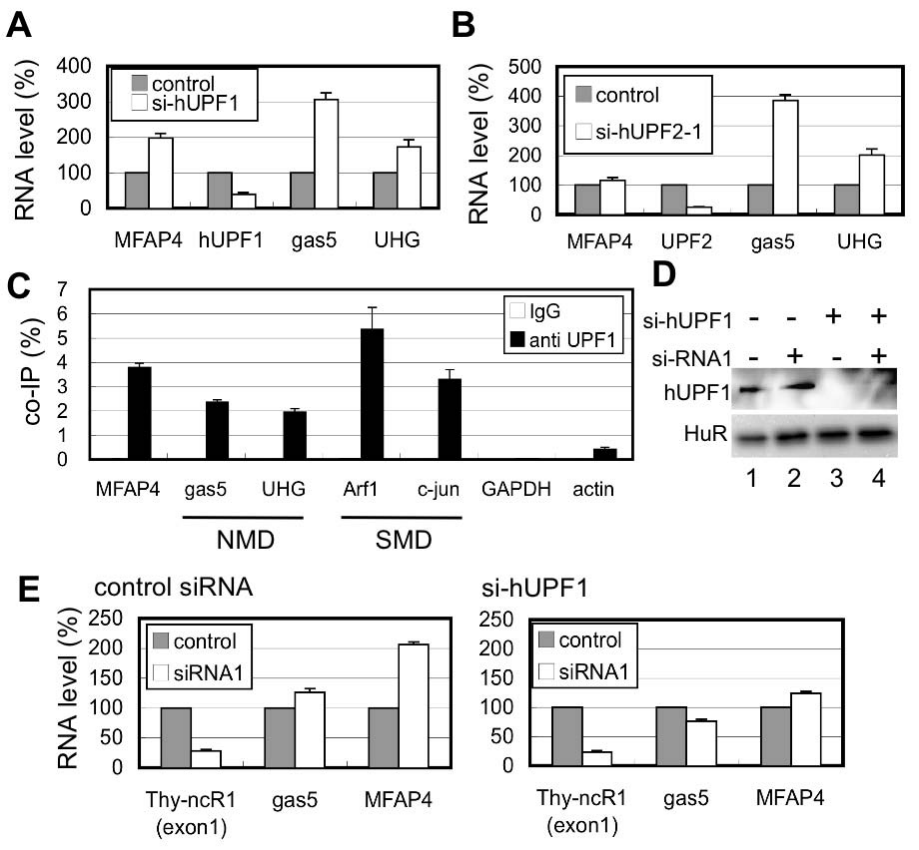
**MFAP4 mRNA level is regulated by a unique hUPF1-dependent pathway**. A, B. Effects of the knockdown of human NMD factors on MFAP4 mRNA. RNAi was conducted using control siRNA, hUPF1-targeted siRNA (si-hUPF1, in A), or hUPF2-targeted siRNA (si-hUPF2-1, in B) in Jurkat cells. C. Co-immunoprecipitation of MFAP4 mRNA from HPB-ALL cell extracts with anti-hUPF1 antibody. Known NMD targets (gas5 and UHG) and SMD targets (Arf1 and c-jun) were used as positive controls. D, E. Double knockdown of Thy-ncR1 and hUPF1. Immunoblot confirmed the depletion of hUPF1 upon treatment with si-hUPF1 in the presence or absence of si-RNA1 against Thy-ncR1 (D). qRT-PCR of MFAP4, gas5, and Thy-ncR1 in Jurkat cells that were treated with each combination of siRNAs shown

The MFAP4 mRNA level increased ~2-fold in response to hUPF1 knockdown, which was an increase similar to that of the known NMD targets, gas5 and UHG, in Jurkat cells (Figure 6A). MFAP4 mRNA appears to be a normal mRNA since no PTCs are predicted according to the sequence information of the cDNA clones in the public databases. This observation suggests that MFAP4 mRNA was unlikely to be subjected to NMD. Intriguingly, hUPF2 RNAi did not change the MFAP4 mRNA level, whereas gas5 and UHG levels were significantly elevated (Figure 6B). These results were confirmed with different siRNAs against hUPF1 or hUPF2 (data not shown). These data indicate that MFAP4 mRNA is degraded by a hUpf1-dependent pathway distinct from NMD.

The RNA-binding protein Staufen 1 binds to a specific stem-loop structure in the 3'UTRs of hUPF1-target mRNAs and recruits hUpf1 to those mRNAs [24]. Our search for such a stem-loop structure failed to identify any typical structure in the 3'UTR of MFAP4 mRNA. Furthermore, Staufen 1 RNAi led to no obvious alteration in the MFAP4 mRNA level in Jurkat cells (data not shown), suggesting that hUpf1 was recruited to MFAP4 mRNA via an unidentified factor. Coimmunoprecipitation using a hUpf1 antibody revealed that MFAP4 mRNA, as well as other known NMD and SMD targets, was efficiently precipitated with the hUpf1 antibody, confirming the association of hUPF1 with MFAP4 mRNA (Figure 6C).

Next, the relationship between hUPF1-dependent MFAP4 mRNA degradation and Thy-ncR1 was examined. As shown in Figure 6D, Thy-ncR1 was depleted in the presence and absence of hUPF1 to examine whether these two factors are involved in different pathways for destabilizing MFAP4 mRNA. The level of MFAP4 mRNA increased 2-fold upon Thy-ncR1 depletion (Figure 6E, control siRNA). However, no obvious further elevation in the MFAP4 mRNA level was observed in the absence of hUPF1 (Figure 6E, si-hUPF1), suggesting that Thy-ncR1 acts to suppress MFAP4 gene expression via the same pathway as that of hUPF1-dependent degradation.

## Discussion

Thy-ncR1 is transcribed from chromosome 1q23.1 within the OR gene cluster. However, Thy-ncR1 was expressed only in the thymus, where the OR genes were not expressed. The expression of the CD1 gene cluster, located 118 kb from Thy-ncR1, was highly correlated with Thy-ncR1 expression in the T-cell lineage, but not in the DC-cell lineage. Thus, the Thy-ncR1 gene was specifically expressed at one stage of T-cell maturation. Expression of Thy-ncR1 may be mechanistically linked to the activation of the CD1 gene cluster expression but not to the expression of the surrounding OR gene cluster.

Our attempts to disrupt Thy-ncR1 function by RNAi were limited, since RNAi may inefficiently degrade the nuclear-localized fraction of Thy-ncR1. The nuclear function of Thy-ncR1 must be examined in future studies to determine whether a mechanistic link exists between the expression of Thy-ncR1 and the CD1 gene cluster. In yeast and fly, ncRNA transcription affects the expression of adjacent genes through transcriptional interference [33,34]. Recently, ncRNA transcription was reported to lead to stepwise chromatin remodeling in the promoter region and activation of the fbp1(+) gene in *Schizosaccharomyces pombe *[35]. These data suggest that Thy-ncR1 may affect CD1 gene expression in cis by a chromosome-associated mechanism that would be difficult to interrupt with Thy-ncR1 RNAi. Since no mouse counterpart has been identified, chromosomal manipulation of the Thy-ncR1 gene in human T-cell lines will be required to explore the nuclear functions of this transcript.

The Thy-ncR1 transcript is likely to be transcribed by RNA polymerase II, as its biogenesis pathway is similar to that of an mRNA, including export to the cytoplasm. Alternative splicing events and putative alternative transcription start sites are able to create at least 24 different Thy-ncR1 isoforms. The intracellular distribution of the Thy-ncR1 isoforms was mostly cytoplasmic, where they were enriched in the small polysome-like fractions. The distribution of the putative target mRNA MFAP4 did not overlap with any of the Thy-ncR1 isoforms in the sucrose gradient fractionation (data not shown), suggesting that the effect of Thy-ncR1 on MFAP4 mRNA stability is indirect and, presumably, mediated by specific regulatory factor(s). Northern hybridization analysis demonstrated that MFAP4 transcript size was unchanged by Thy-ncR1 depletion. The unspliced MFAP4 pre-mRNA level remained constant after Thy-ncR1 depletion (data not shown). These data strongly suggest that Thy-ncR1 depletion results in the destabilization of the mature MFAP4 mRNA rather than affecting transcription or splicing regulation. A sequence comparison of Thy-ncR1 with MFAP4 mRNA revealed that a short pyrimidine stretch (~30 nt) located downstream of the MFAP4 mRNA initiation codon is similar to a sequence in exon 3 of Thy-ncR1 (Additional file 1, Fig. S6). This similarity suggests the intriguing possibility that Thy-ncR1 acts to capture an MFAP4 mRNA stabilizing factor recognizing the pyrimidine-rich stretch. This model would explain how Ex1-3 and Ex1-2-3, but not Ex1-4-5, can influence MFAP4 mRNA levels. The various splicing isoforms may play distinct regulatory roles in the cytoplasm, one of which is to regulate MFAP4 mRNA stability.

MFAP4 mRNA was destabilized via a hUPF1-dependent pathway in Jurkat cells. RNAi depletion of hUPF1 stabilized MFAP4 mRNA, and a hUPF1 antibody co-immunoprecipitated MFAP4 mRNA. Translation arrest with cycloheximide also stabilized MFAP4 mRNA, indicating that hUPF1 is involved in a translation-dependent mRNA degradation pathway (data not shown). The evidence that MFAP4 mRNA possesses no putative PTCs and that neither hUPF2 nor Staufen 1 is involved in MFAP4 mRNA destabilization indicates that a novel hUPF1-dependent mRNA decay pathway regulates the MFAP4 mRNA level in Jurkat cells. Identification of a new hUPF1 adaptor may be the next step in defining the molecular mechanism of this mRNA degradation pathway.

Our results here demonstrated that Thy-ncR1 acts to reduce the level of MFAP4 mRNA. A double-knockdown experiment suggested that the Thy-ncR1-dependent pathway overlaps with the hUPF1-dependent pathway for MFAP4 mRNA degradation. We confirmed that hUPF1 association with MFAP4 mRNA is not altered in the presence or absence of Thy-ncR1 and that Thy-ncR1 isoforms were poorly co-immunoprecipitated with anti-hUPF1 antibody, suggesting that Thy-ncR1 is not directly involved in hUPF1 binding to MFAP4 mRNA (data not shown). Thy-ncR1 may indirectly control a cytoplasmic regulatory factor that modulates the activity of hUPF1 or other factors involved in this decay pathway.

MFAP4 encodes a polypeptide that contains a fibronectin-like domain for cell adhesion. A recent report revealed that MFAP4 facilitates the *ex vivo *expansion of hematopoietic stem cells, suggesting that MFAP4 acts as an angiopoietin-like growth factor [36]. The specific physiological role of MFAP4 in immature T cells remains to be clarified. Elucidation of the physiological role of MFAP4 may also reveal the significance of the regulation of MFAP4 gene expression by the thymus-specific riboregulator, Thy-ncR1.

## Conclusions

In summary, our results show that Thy-ncR1 ncRNA is a functional cytoplasmic "riboregulator" at one specific stage of T-cell maturation. Thy-ncR1 controls the abundance of MFAP4 mRNA, which is degraded via an uncharacterized hUPF1-dependent degradation pathway.

## Methods

### Cell lines

HPB-ALL and Jurkat cells were cultured in RPMI medium supplemented with 10% fetal bovine serum (FBS) and grown in a humidified incubator (5% CO_2_) at 37°C. Jurkat cells were plated at a density of 2-3 × 10^5 ^cells/ml and used in experiments after a 24 h incubation. HPB-ALL cells were plated at a density of 1 × 10^6 ^cells/ml and used in experiments after a 48 h incubation. Induction of CD14^+ ^cell differentiation into dendritic cells (DC) was performed as described previously with some modifications [32]. Briefly, CD14^+ ^umbilical cord blood monocytes were enriched using a magnetic cell sorter and then cultured in alpha-MEM containing 10 ng/ml of each of recombinant human GM-CSF and IL-4 for 7 days. Lipopolysaccharide (1 μg/ml) was added to the culture during the last 24 h to complete the maturation of the DC.

### RNA analyses

Total RNA was prepared using Trizol (Invitrogen) or Sepasol (Nacalai). RNase protection assays were performed as described previously [37]. PCR fragments that cover each ncRNA region were cloned into the pGEM-T Easy vector (Promega), followed by digestion with an appropriate restriction enzyme for *in vitro *transcription. Total RNA (3-10 μg) was hybridized to a ^32^P-labeled antisense RNA probe that was synthesized using the T7 or SP6 RNA polymerase (TaKaRa). RNase A/T1 digestion removed single-stranded RNA. The protected RNA fragments were separated by polyacrylamide gel electrophoresis (PAGE) on an 8% gel containing 7 M urea. Radioactive RNA bands were visualized using Fuji BAS3000 Bioimaging Analyzer. For northern hybridization, total RNA was run on an 8% PAGE gel containing 7 M urea followed by electroblotting onto a Hybond N^+ ^nitrocellulose membrane (GE Healthcare Life Sciences). The blotted RNAs were covalently fixed to the membrane by UV crosslinking. Radiolabeled DNA probes were synthesized from cDNA fragments using a DNA labeling Kit (Takara) and hybridized in ExpressHyb™ Hybridization Solution (Clontech) at 50°C overnight. The membrane was washed, and radiolabeled RNA bands were visualized using Fuji BAS3000 Bioimaging Analyzer.

Quantitative reverse transcription polymerase chain reaction (qRT-PCR) was performed as described [18]. Briefly, total RNA (1 μg) was reverse transcribed using the QuantiTect reverse transcription kit (Qiagen). Primers were designed using Primer3 software http://www-genome.wi.mit.edu/ftp/distribution/software/ and purchased from Invitrogen. Aliquots of cDNA were subjected to real-time PCR performed using LightCycler 480 SYBR Green I Master (Roche Diagnostics) according to the manufacturer's protocol. No amplification was observed when reverse transcriptase was omitted. Primer sequences for qRT-PCR are provided in Additional file 1, Table S1.

### RNAi

Stealth siRNAs for Thy-ncR1, hUPF1, and hUPF2 were synthesized by Invitrogen, and an siRNA for hUPF1 was synthesized by Proligo. A Stealth™ RNAi Negative Control Kit (Invitrogen) and Silencer^® ^Negative Control #1 siRNA (Ambion, Inc.) were used for negative controls. Prior to transfection, cells were cultured in medium containing 10% FBS without antibiotics. Subsequently, 2 × 10^6 ^Jurkat or HPB-ALL cells were resuspended in 100 μL Nucleofector Solution-V (Amaxa) and mixed with 400 pmol stealth siRNA for Thy-ncR1 or 200 pmol siRNA for hUPF1 and hUPF2. The siRNA transfection used the G-010 protocol according to the manufacturer's instructions (Amaxa). The cells were plated in RPMI1640 medium containing 10% FBS. After 48 h, total RNA was prepared using an RNeasy kit (Qiagen). Sequences of the siRNAs are provided in Additional file 1, Table S2.

### DNA Microarray

HPB-ALL cells were nucleofected with control siRNA, siRNA1, siRNA3-1, or siRNA3-2, and incubated for 48 h. Total RNA was then prepared and labeled with Cy3. Samples were hybridized to a Human Oligo Microarray (G4112F, Agilent) according to the manufacturer's protocol. Arrays were scanned with a G2565BA Microarray Scanner System (Agilent), and the resulting data were analyzed using the GeneSpring GX software (Agilent). The microarray analysis to monitor human non-protein coding transcripts was conducted as described previously [26]. The raw data are available in Center for Information Biology Gene Expression Database (CIBEX; http://cibex.nig.ac.jp)(accession number: CBX142).

## Authors' contributions

KA and TH designed the experiments. KA, MS, and TH carried out the experiments shown in Figure 1, 2, 3, 4, 5, 6. AH, SN, and SK prepared the RNA samples used in Figure 1. TY performed the microarray analysis whose data was used in Figure 5. KA and TH wrote the manuscript. All authors read and approved the final manuscript.

## Supplementary Material

Additional file 1 **Supplemental Figures**. Supplemental Figure S1. The expression profiles of 10 ncRNAs that are predominantly expressed in thymus. qRT-PCR was conducted to measure the levels of each ncRNA in 11 human tissues (1, brain; 2, thymus; 3, heart; 4, lung; 5, liver; 6, spleen; 7, kidney; 8, testis; 9, placenta; 10, prostate; and 11, skeletal muscle). The levels in thymus were defined a 100%. Supplemental Figure S2. A. RNase protection assays to detect transcripts derived from exon 1 and exon 2. B. Confirmation of the absence of the OR10R2 transcript in the thymus. An RNase protection assay was conducted to detect OR10R2 mRNA using a sense probe (S) and Thy-ncR1 using an antisense probe (AT). The Thy-ncR1 transcripts detected by the AT probe are shown by the arrows. Detection of additional isoforms may indicate differential usage of 5' end start sites. Supplemental Figure S3. Genomic structure of the CD1 gene cluster, the Thy-ncR1 locus, and neighboring genes in human (above) and their counterparts in mouse. Supplemental Figure S4. Polysome profiles of Thy-ncR1 isoforms. The cell extract prepared from HPB-ALL cells treated with cycloheximide was subjected to 15-45% sucrose density gradient centrifugation (left panels). Sucrose density gradient centrifugation was carried out with an HPB-ALL cell extract treated with 50 mM EDTA (right panels). RNA prepared from each fraction was used for qRT-PCR. The amount of transcript in each fraction relative to the total RNA was calculated and shown in the bottom panel. Supplemental Figure S5. A. Amino acid sequences encoded by potential ORFs in Thy-ncR1 isoforms and their conservation among four primate species. B. Potential ORFs in the gas5 and UHG ncRNAs. Supplemental Figure S6. Significant sequence similarity between a region in the MFAP4 mRNA and the Thy-ncR1 exon 3 (red letters). Supplemental table S1-Primer Sequences using for RT-PCR. Supplemental table S2-siRNA Sequences Click here for file
