# Supplementary material for: A thymus-specific noncoding RNA, Thy-ncR1, is a cytoplasmic riboregulator of MFAP4 mRNA in immature T-cell lines
Source: BMC Mol Biol. 2010 Dec 16;11:99. doi: 10.1186/1471-2199-11-99 (PMC3023731; doi:10.1186/1471-2199-11-99)
Supplement: Additional file 1 — Supplemental Figures. Supplemental Figure S1. The expression profiles of 10 ncRNAs that are predominantly expressed in thymus. qRT-PCR was conducted to measure the levels of each ncRNA in 11 human tissues (1, brain; 2, thymus; 3, heart; 4, lung; 5, liver; 6, spleen; 7, kidney; 8, testis; 9, placenta; 10, prostate; and 11, skeletal muscle). The levels in thymus were defined a 100%. Supplemental Figure S2. A. RNase protection assays to detect transcripts derived from exon 1 and exon 2. B. Confirmation of the absence of the OR10R2 transcript in the thymus. An RNase protection assay was conducted to detect OR10R2 mRNA using a sense probe (S) and Thy-ncR1 using an antisense probe (AT). The Thy-ncR1 transcripts detected by the AT probe are shown by the arrows. Detection of additional isoforms may indicate differential usage of 5' end start sites. Supplemental Figure S3. Genomic structure of the CD1 gene cluster, the Thy-ncR1 locus, and neighboring genes in human (above) and their counterparts in mouse. Supplemental Figure S4. Polysome profiles of Thy-ncR1 isoforms. The cell extract prepared from HPB-ALL cells treated with cycloheximide was subjected to 15-45% sucrose density gradient centrifugation (left panels). Sucrose density gradient centrifugation was carried out with an HPB-ALL cell extract treated with 50 mM EDTA (right panels). RNA prepared from each fraction was used for qRT-PCR. The amount of transcript in each fraction relative to the total RNA was calculated and shown in the bottom panel. Supplemental Figure S5. A. Amino acid sequences encoded by potential ORFs in Thy-ncR1 isoforms and their conservation among four primate species. B. Potential ORFs in the gas5 and UHG ncRNAs. Supplemental Figure S6. Significant sequence similarity between a region in the MFAP4 mRNA and the Thy-ncR1 exon 3 (red letters). Supplemental table S1-Primer Sequences using for RT-PCR. Supplemental table S2-siRNA Sequences [file 1471-2199-11-99-S1.PPT]

## Slide 1
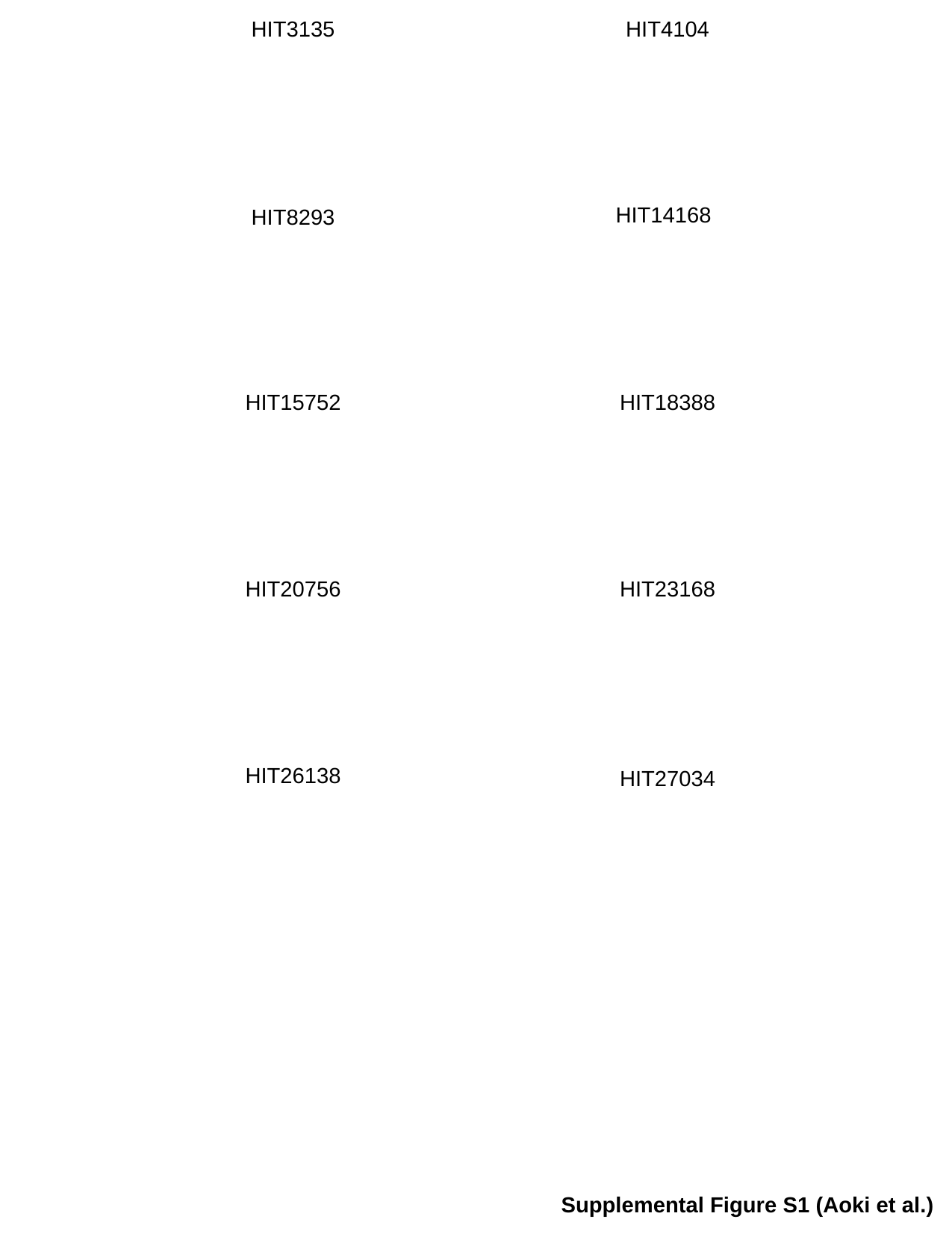

HIT3135
HIT4104
HIT14168
HIT8293
HIT15752
HIT18388
HIT20756
HIT23168
HIT26138
HIT27034
Supplemental Figure S1 (Aoki et al.)

## Slide 2
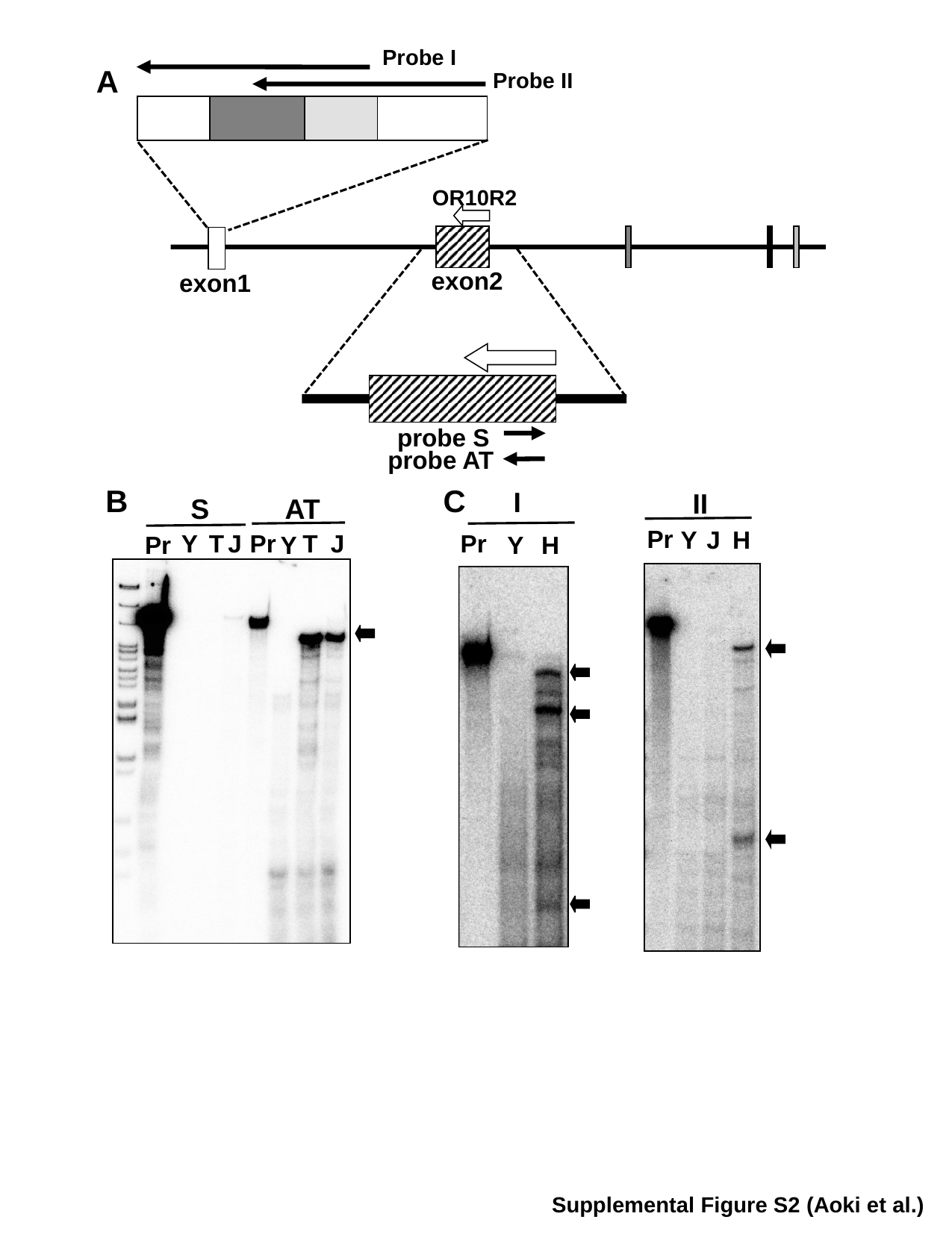

Probe I
A
Probe II
OR10R2
exon2
exon1
probe S
probe AT
B
C
I
II
S
AT
Pr
Y
J
H
Y
T
J
Pr
T
J
Pr
Pr
Y
Y
H
Supplemental Figure S2 (Aoki et al.)

## Slide 3
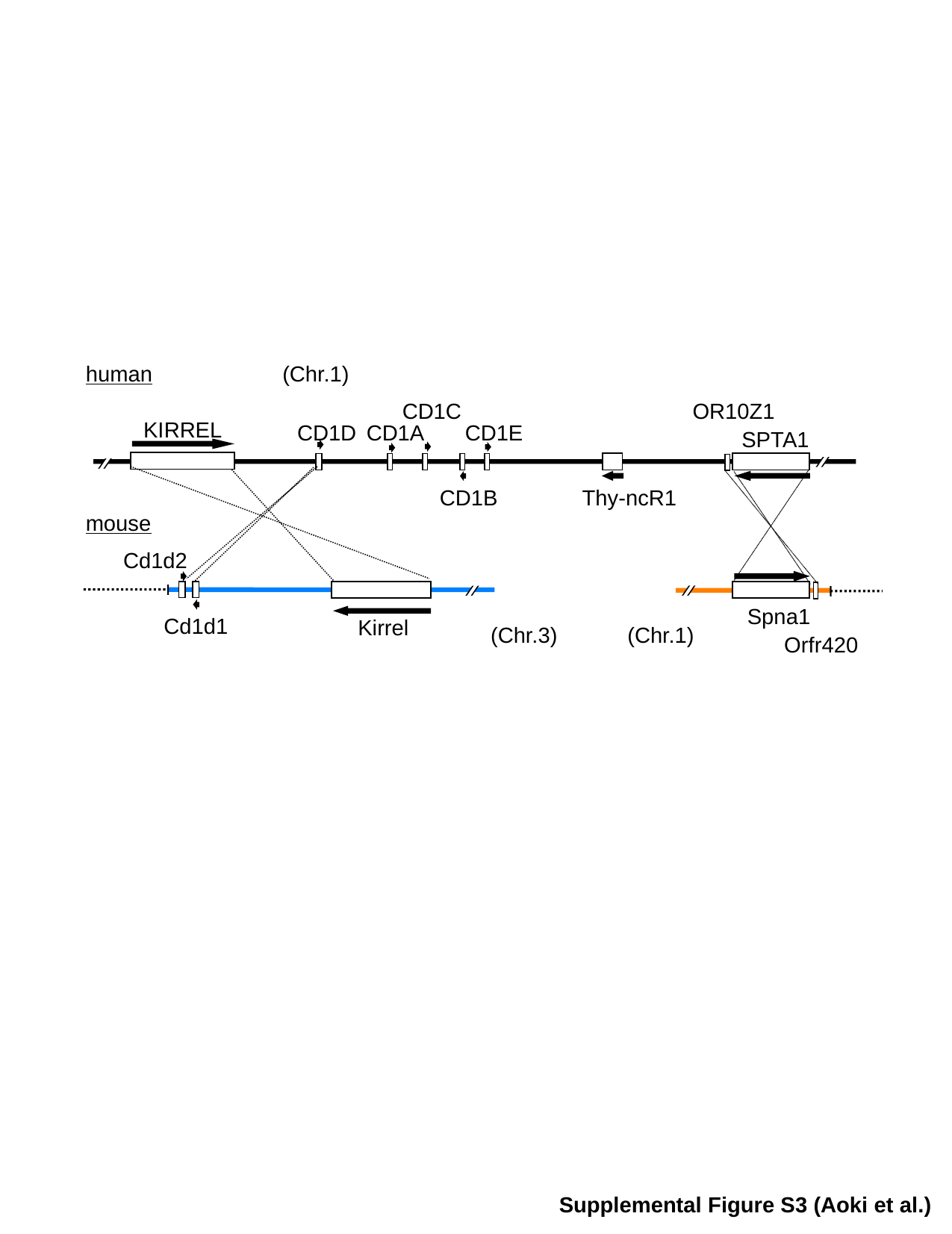

human
(Chr.1)
CD1C
OR10Z1
KIRREL
CD1D
CD1A
CD1E
SPTA1
CD1B
Thy-ncR1
mouse
Cd1d2
Spna1
Cd1d1
Kirrel
(Chr.3)
(Chr.1)
Orfr420
Supplemental Figure S3 (Aoki et al.)

## Slide 4
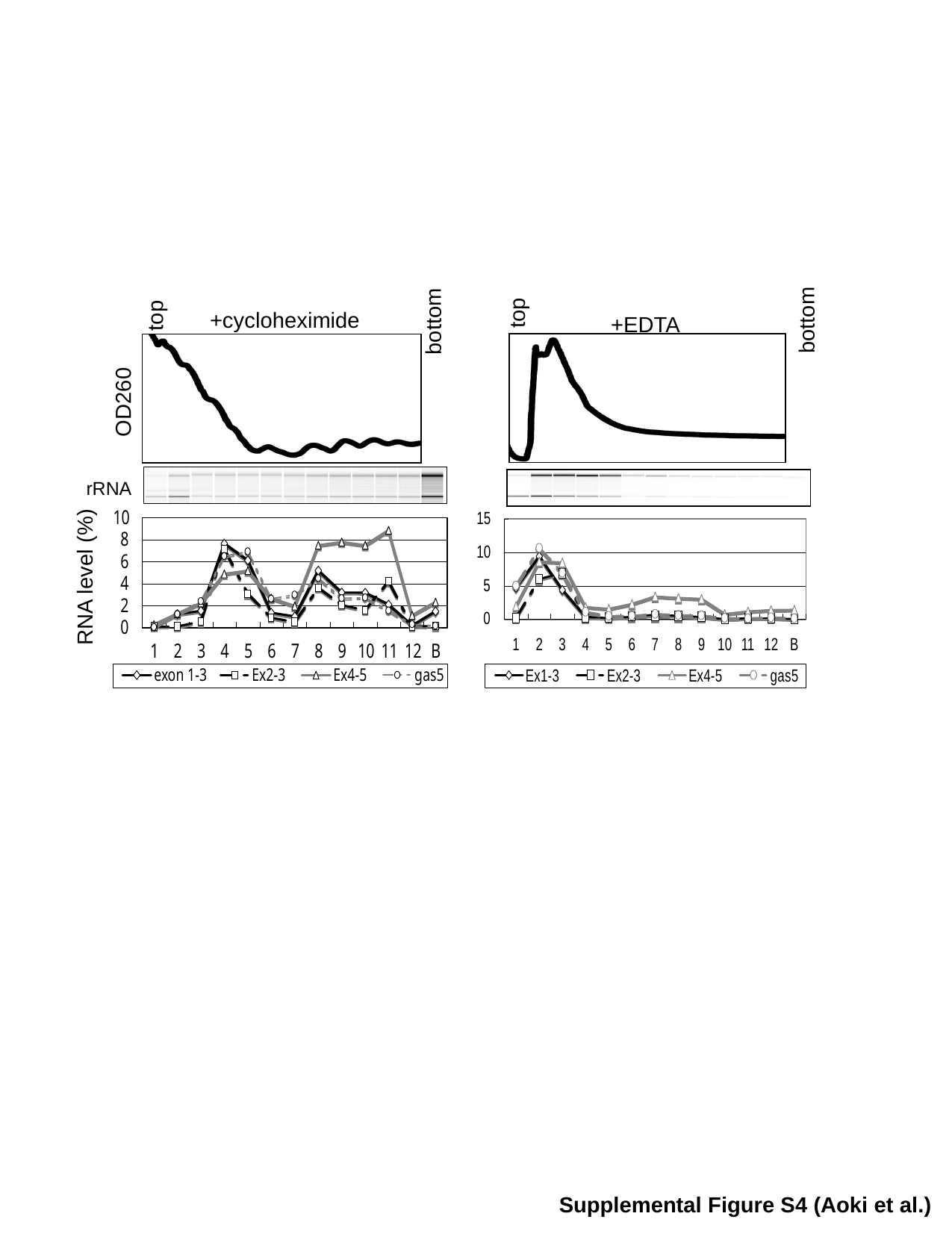

top
top
+cycloheximide
bottom
bottom
+EDTA
OD260
rRNA
RNA level (%)
Supplemental Figure S4 (Aoki et al.)

## Slide 5
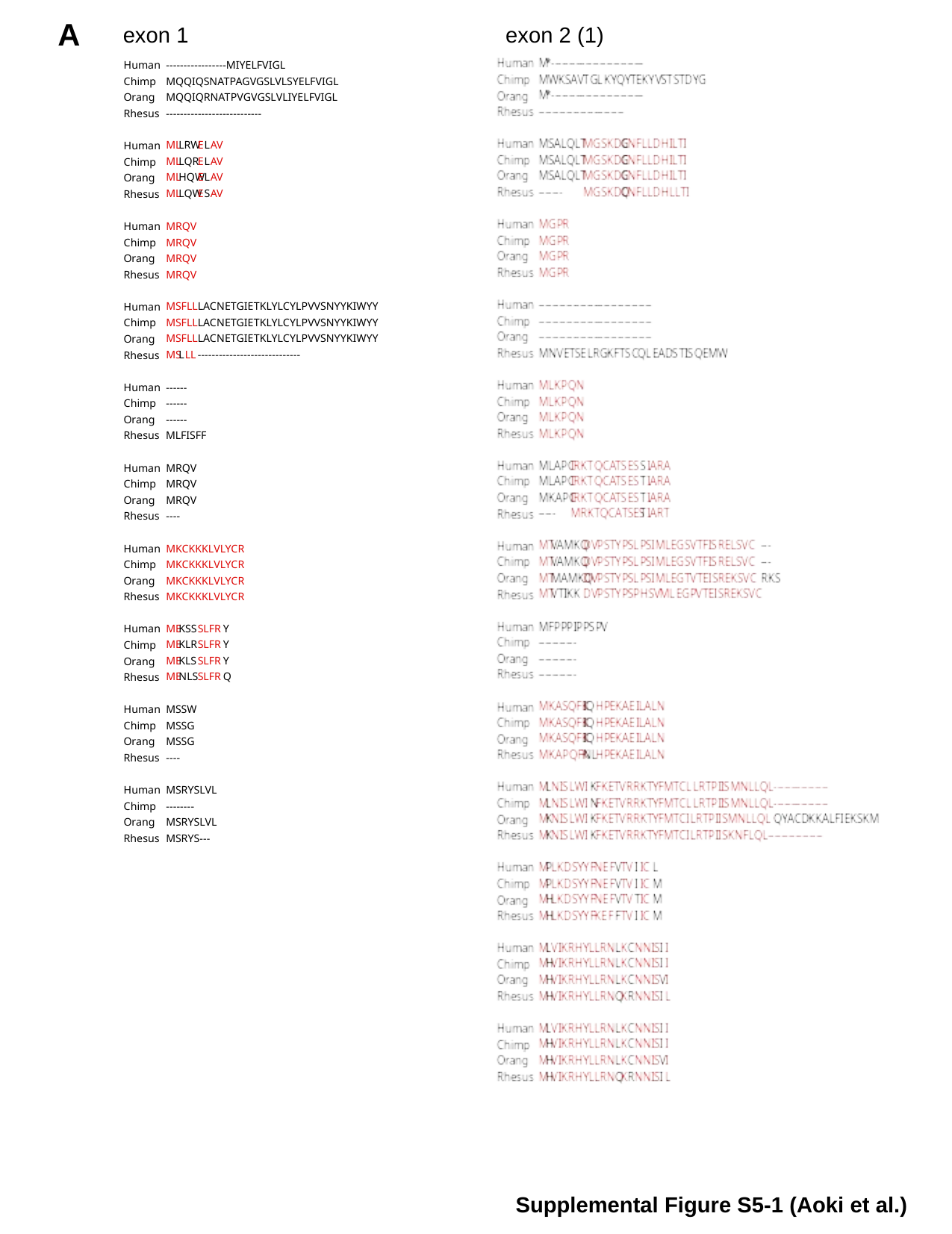

A
exon 1
exon 2 (1)
Supplemental Figure S5-1 (Aoki et al.)

## Slide 6
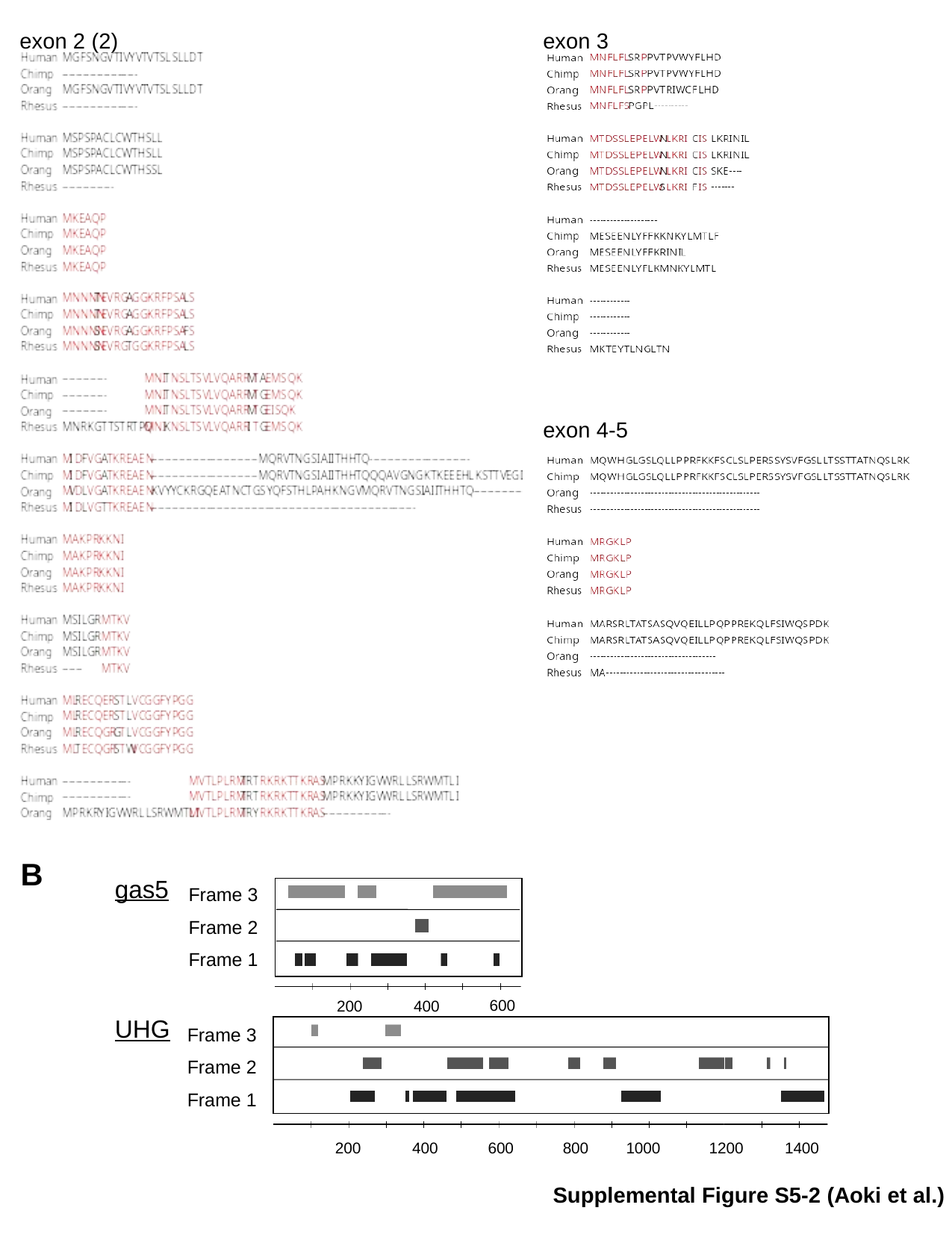

exon 2 (2)
exon 3
exon 4-5
B
gas5
Frame 3
Frame 2
Frame 1
600
200
400
UHG
Frame 3
Frame 2
Frame 1
200
400
600
800
1000
1200
1400
Supplemental Figure S5-2 (Aoki et al.)

## Slide 7
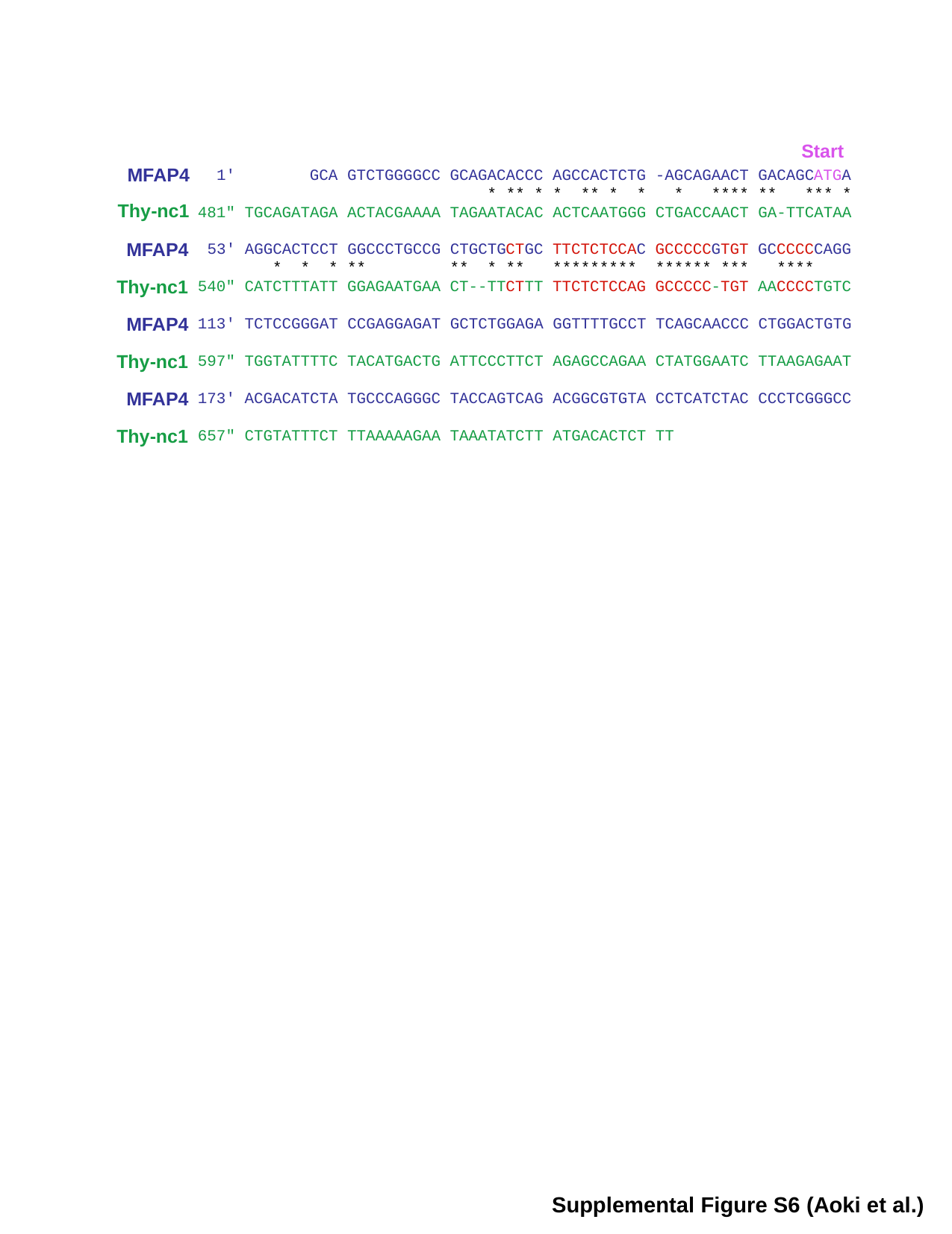

Start
MFAP4
 1' GCA GTCTGGGGCC GCAGACACCC AGCCACTCTG -AGCAGAACT GACAGCATGA
 * ** * * ** * * * **** ** *** *
 481" TGCAGATAGA ACTACGAAAA TAGAATACAC ACTCAATGGG CTGACCAACT GA-TTCATAA
 53' AGGCACTCCT GGCCCTGCCG CTGCTGCTGC TTCTCTCCAC GCCCCCGTGT GCCCCCCAGG
 * * * ** ** * ** ********* ****** *** ****
 540" CATCTTTATT GGAGAATGAA CT--TTCTTT TTCTCTCCAG GCCCCC-TGT AACCCCTGTC
 113' TCTCCGGGAT CCGAGGAGAT GCTCTGGAGA GGTTTTGCCT TCAGCAACCC CTGGACTGTG
 597" TGGTATTTTC TACATGACTG ATTCCCTTCT AGAGCCAGAA CTATGGAATC TTAAGAGAAT
 173' ACGACATCTA TGCCCAGGGC TACCAGTCAG ACGGCGTGTA CCTCATCTAC CCCTCGGGCC
 657" CTGTATTTCT TTAAAAAGAA TAAATATCTT ATGACACTCT TT
Thy-nc1
MFAP4
Thy-nc1
MFAP4
Thy-nc1
MFAP4
Thy-nc1
Supplemental Figure S6 (Aoki et al.)

## Slide 8
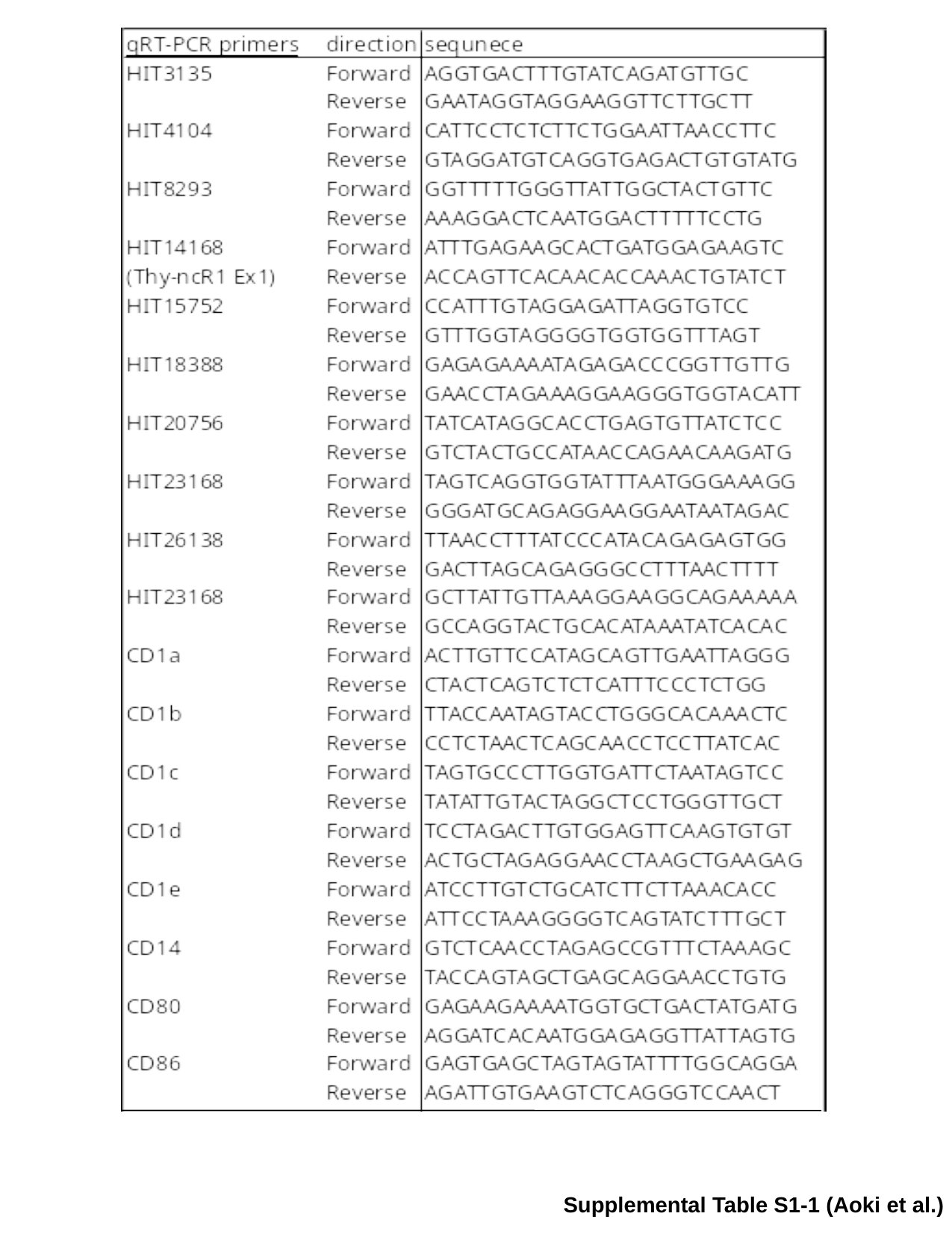

Supplemental Table S1-1 (Aoki et al.)

## Slide 9
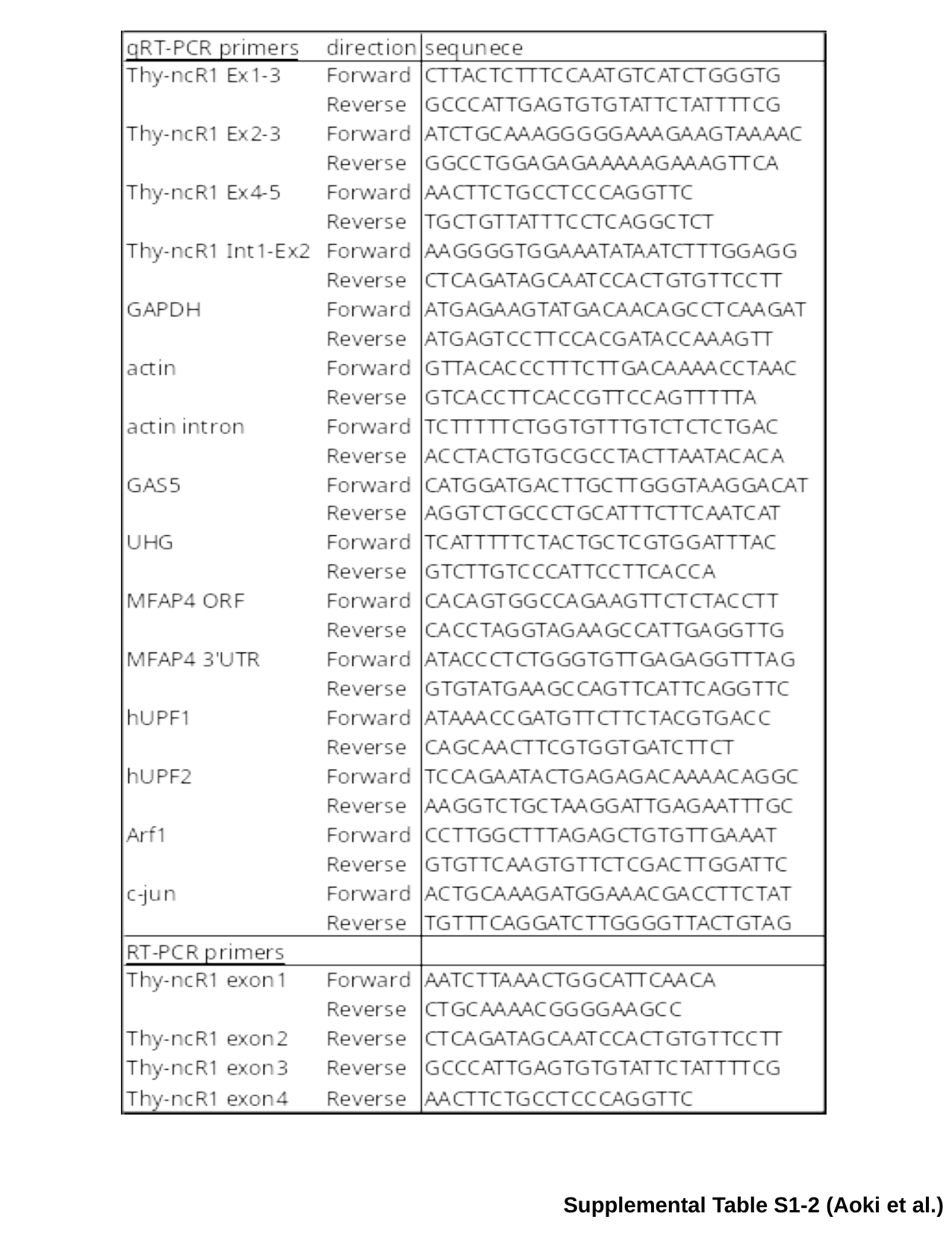

Supplemental Table S1-2 (Aoki et al.)

## Slide 10
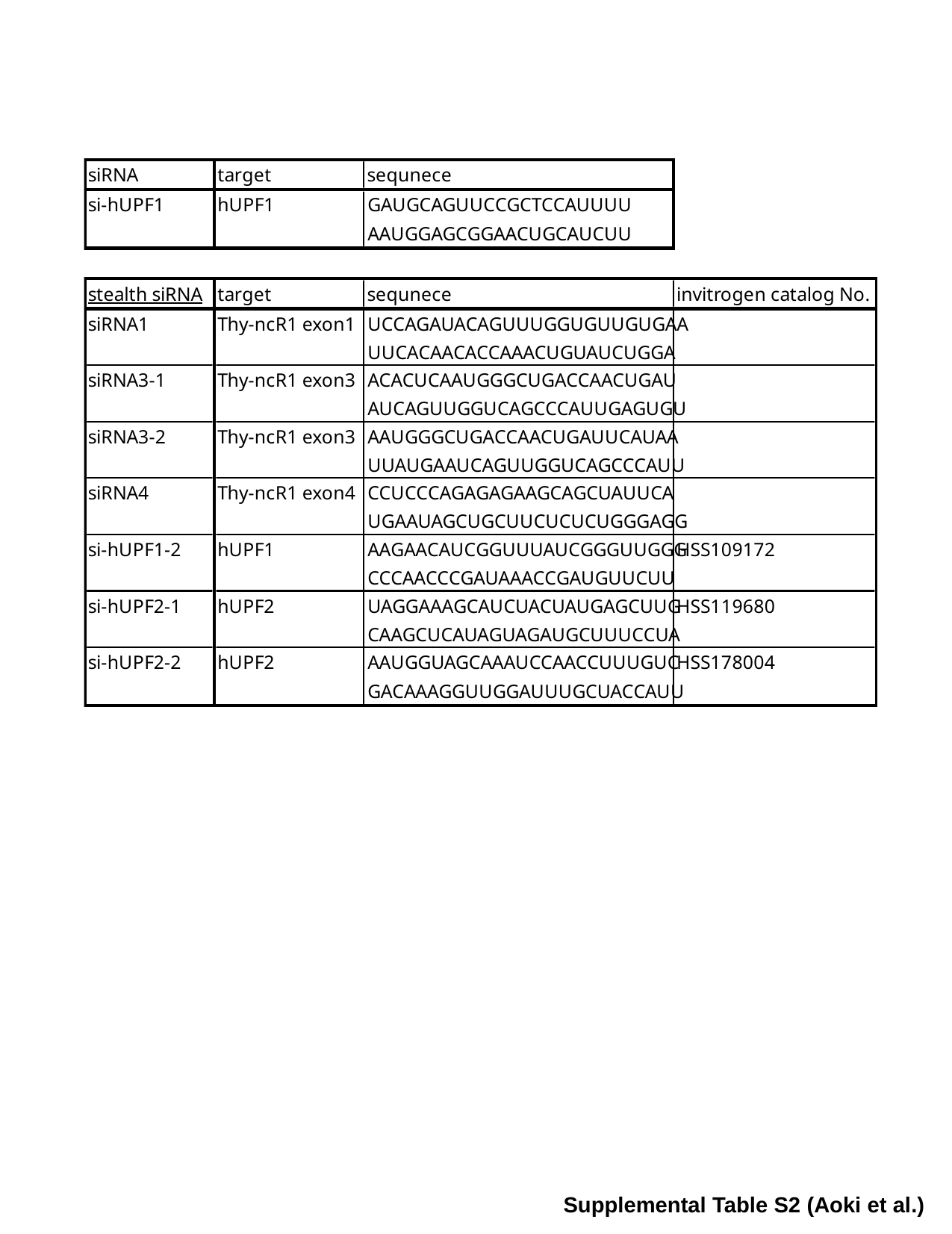

Supplemental Table S2 (Aoki et al.)
